# Understanding and integrating the needs and preferences of people living with dementia in the inpatient setting: a qualitative study

**DOI:** 10.1186/s12877-025-05932-7

**Published:** 2025-05-15

**Authors:** Alyse Lennox, Denise Goodwin, Felicity Leavold, Renae Nicol, Velandai Srikanth, Darshini Ayton, Madeleine Berends, Myra Thiessen, Daphne Flynn, Chris Moran

**Affiliations:** 1 Monash Sustainable Development Institute, BehaviourWorks Australia, Monash University, Melbourne, Australia; 2 Department of Nursing, Peninsula Health, Melbourne, Australia; 3 National Centre for Healthy Ageing, Melbourne, Australia; 4 Department of Geriatric Medicine, Peninsula Health, Melbourne, Australia; 5 Health and Social Care Unit, School of Public Health and Preventive Medicine, Monash University, Melbourne, Australia; 6 Consumer representative, Melbourne, Australia; 7 Art, Design and Architecture, Monash University, Melbourne, Australia; 8 Department of Aged Care, Alfred Health, Melbourne, Australia; 9 School of Public Health and Preventive Medicine, Monash University, Melbourne, Australia

**Keywords:** Dementia, Qualitative research, Person-centred care, Staff, Support people

## Abstract

**Background:**

People living with dementia (PLWD) have poorer outcomes than cognitively normal people when admitted to hospital. One reason for this difference is related to the challenges in learning and integrating the needs and preferences of PLWD into clinical care. We aimed to obtain a range of perspectives on the challenges in supporting PLWD in hospital and explore opportunities for improvement.

**Methods:**

Using an exploratory qualitative study design, we conducted interviews with nine people supporting PLWD (current / ex-spouses or children of PLWD) and 11 nursing, medical and allied health staff members at a single Australian hospital. Data were thematically analysed using a framework approach.

**Results:**

Participants described how best practice in supporting PLWD included understanding and integrating patient needs and highlighted the importance of family and the multidisciplinary team working in partnership. A number of factors inhibiting quality care provision were described, including uncertainty around responsibility for communicating with families to understand needs of PLWD; unsuitable tools; lack of opportunities for families to communicate with staff; and, resource and environmental constraints. Participants discussed potential for improvement, emphasising the need for a pre-emptive, rather than reactive solution. They expressed support for the idea of a ‘hospital admission kit’, containing both information about PLWD and their familiar items. Implementation considerations were also noted, with various perspectives on the timing of initiation, updating, responsible person(s), format, content and how it should be incorporated into clinical workflows.

**Conclusions:**

We found that hospital staff and those supporting PLWD felt that integrating the needs and preferences of PLWD into hospital care was important. The concept of a pre-prepared ‘toolkit’ that was ready in the case of a need to attend hospital was felt to be valid and potentially helpful. More work is required to design aspects such as format, content and the workflows needed to generate accountability and reliability in creating, updating and incorporating it into hospital care.

**Clinical trial number:**

Not applicable.

**Supplementary Information:**

The online version contains supplementary material available at 10.1186/s12877-025-05932-7.

## Background

There is a large body of evidence reporting that people living with dementia (PLWD) who are admitted to hospital have poorer outcomes than inpatients without cognitive impairment [1, 2]. These poorer outcomes include patient-centred outcomes such as greater complications, greater loss of functional independence and increased mortality, as well as process measures such as longer hospital stays and greater healthcare system costs [1]. Although involving patients in their own care is well-established to improve outcomes [3] and is a core part of modern clinical care [4–11], it is often done informally, inconsistently and is poorly communicated to others [4, 6, 7, 12–16].

There are few tools to support health services staff to learn the needs and preferences of PLWD [6, 13, 14, 17] and existing tools are often limited in routine use by a lack of clear guidance about how they should be operationalised, with previous work highlighting that there is often poor understanding of how relevant information should be generated, updated and incorporated into clinical care [18, 19]. One reason for this lack of understanding is that many existing tools have been designed with a focus on either the perspective of those supporting a PLWD at home or hospital staff using such tools [18, 19]. We are unaware of a tool that has been designed by both groups in partnership.

To help develop a tool to facilitate a process for identifying the needs and preferences of PLWD when admitted to hospital, we aimed to explore various perspectives on understanding and integrating the needs of PLWD when they are admitted to hospital, as well as capture opportunities for improvement and potential solutions. The broad research questions for each group were: (1) what information is important for hospital staff to know when providing hospital care for a PLWD? and (2) what processes would help staff identify and use this information to meet the needs and preferences of PLWD?

## Methods

### Study design

This was an exploratory qualitative study designed to understand various perspectives on understanding and integrating the needs and preferences of PLWD when they are admitted to hospital and was underpinned by a phenomenological approach [20].

### Study population and setting

This project is part of the Living Labs Research Program of the National Centre for Healthy Ageing (ncha.org.au), a partnership between Monash University and Peninsula Health, a major metropolitan health service in Victoria, Australia. The study was conducted at the Acute Care of the Elderly (ACE) Unit at Frankston Hospital, an acute hospital with 454 beds within Peninsula Health. The ACE unit is co-located on an oncology ward and can provide acute medical care for a maximum of 30 older people at any time. Frankston Hospital is an acute university-affiliated tertiary metropolitan teaching hospital with 454 beds. Guided by the concept of ‘information power’, i.e. the more information a sample holds that is relevant to the study aims, the lower number of participants is required [21], we included a small number of staff across a range of relevant disciplines with varying levels of seniority. We aimed to recruit 10 people admitted to the Acute Care of Elderly (ACE) Unit at Peninsula Health with a known history of dementia, 10 people who are supporting someone living with dementia admitted to the ACE Unit and 10 hospital clinicians caring for PLWD on the ACE Unit. Hospital clinicians were purposively selected to ensure a variety of perspectives based on professional role (nursing, medical and allied health) and levels of seniority (junior to senior positions). This selection process aimed to capture a broad range of experiences in caring for PLWD. Hospital clinicians were eligible to participate if they had worked in the ACE Unit for more than 7.5 h in the 2 months preceding the interview.

### Recruitment

Potential participants were invited to participate in a semi-structured interview by Peninsula Health clinical researchers, who had the expertise to determine whether PLWD had the capacity to consent to participate in an interview. These clinicians (senior nurse and geriatrician) had extensive training and expertise in working with PLWD. Capacity to participate in an interview was assessed via a clinical interview if the PLWD was alert enough to participate. Staff members were invited to participate by either the nurse lead of the project (FL) or the medical lead (CM). PLWD and those supporting them were approached to participate either face to face or over the telephone by the cognition nurse consultant (RN) or the medical lead (CM), not their usual treating clinician. Research aims and procedures were outlined in an Explanatory Statement given to all participants prior to the interview and all participants provided voluntary informed consent.

### Data collection

A trained and experienced female researcher (AL) conducted all interviews via telephone or Zoom between July and December 2022. The interviewer was a Senior Research Officer with a background in psychology (BPsych (Hons)), who had no lived experience of working with or caring for someone with dementia, nor did they have any prior relationships with the participants. Participants were informed of the interviewer’s professional role and role in the study. No interviewer characteristics were reported. The interview guide (See Appendix 1) was developed to address the aims of the study. While this was not pilot tested, it was reviewed by the broader research team, including those working with PLWD and a consumer with lived experience of caring for a PLWD. Questions for support people explored PLWD’s hospital admission experiences; existing home routines and translation to the hospital environment; support provided by hospital staff; preparation for hospital admission; and communication about needs and preferences. Questions for staff explored tools to understand PLWD’s needs and preferences; integration of needs and preferences into care; and training and support. Both groups of participants were also asked to consider the usefulness of a ‘hospital admission kit’, similar to a ‘go bag’ used in maternal health settings, as a potential solution. This was defined broadly with the intention to gather initial concept ideas before progressing to a separate co-design phase. Therefore, it was not designed to constitute a comprehensive exploration of this concept. Probes were used throughout the interviews to encourage participants to explain or expand on their responses.

All interviews were conducted in English and the mean interview time was 36 min (range: 23–59). No non-participants were present during the interviews. Interviews were digitally audio-recorded and transcribed verbatim using an online transcription service. The first author reviewed the transcripts for accuracy. Participants were able to request a copy of their transcript for review, however no one did.

### Data analysis

Interview transcripts were loaded into a computer-assisted qualitative data analysis software program (NVivo 20, QSR International Pty Ltd 2022, Doncaster). Thematic analysis was conducted by the first author using a framework approach [22]. This process commenced with repeatedly reading the transcripts to ensure familiarisation. Repetitions in text and meaning were identified and coded. Through an inductive and iterative process, key themes and subthemes were formed based on patterns and connections within and between transcripts. Similar themes were clustered. The resulting framework was applied to the original transcripts to check for meaningful links and connections and adjustments were made to ensure accurate representation. Direct quotations were used to illustrate key themes. Participants did not provide feedback on the findings.

## Results

A total of 20 interviews were conducted. This included 11 healthcare professionals, including nurses (*n* = 9), allied health staff (*n* = 1) and medical staff (*n* = 1). A total of 13 people supporting a PLWD initially agreed to participate in the study with nine ultimately participating. Most PLWD who were admitted to the ward during the study period were too confused (many with superimposed delirium) to be able to concentrate for long enough to understand the purpose of the study and consent. Two PLWD were deemed potentially appropriate to participate in the study but did not consent to do so due to becoming frequently unwell. All staff members who were approached, agreed to participate. For staff, years of experience in their current role ranged from one month to 10 years. Support people included spouses (*n* = 4), ex-partners (*n* = 1) and children (*n* = 4) of those living with dementia. Among the PLWD that they were supporting, experience of supporting symptoms of dementia ranged from 8 weeks to 8 years. The majority of participants reported that the person that they were supporting was living at home (*n* = 6) prior to admission, with the remainder in a nursing home, respite or a serviced retirement village. However, one participant noted that the PLWD they were supporting would be moving into a nursing home following the recent hospital admission.

Three main themes and multiple sub-themes were identified and are described below. Additional detail and quotations supporting the themes and sub-themes are presented in Table 1.

**Table 1:** Themes, sub-themes and supporting quotations

Theme (Sub-theme) - Example codes	Supporting quotations
**Key principles of quality care for PLWD in hospital**	
(Understanding and integrating needs improves patient care) - Understanding and integrating needs - Communicating needs to staff	*“That’s [integrating needs and preferences into care] your starting point. Everything else is extra… if you understand your patients’ triggers*,* you know what to avoid”.* (Nursing_7 months in current role_#2) *“It’s [understanding and integrating needs and preferences into care] incredibly important*,* because a lot of what we do is so reactive here… So*,* I think it’s very clear that it improves patient care across the board”.* (Medical_6 months in current role_#8) *“They [PLWD] just go back to whatever they were doing in their life*,* like some of them like to clean*,* and they want to clean the whole ward. Some are doing the laundry kind of things*,* the folding… They’re just happy to do those kind of things”.* (Nursing_10 years in current role_#6) *“I was just thinking this morning*,* maybe I’ll take some juice in with me because he’s always had his tablets with a glass of orange juice*,* because that’s how he likes it. And I think with dementia*,* a lot of routine is good for someone with dementia and I think maybe he’s out of that comfort zone at the moment of having a routine… He’s always had most of his tablets at breakfast time with his breakfast and then one tablet at lunchtime and one at tea time. I mean when the nurse rang me the other night and said he won’t take his medication*,* that was 9 o’clock. It was quite late for that tablet”.* (Wife of PLWD_#4) *“It’s not about trying to stop things happening. It’s about how can we respond and react in a way that minimises harm for both the patient and the staff”.* (Nursing_8 months in current role_#10)
(Partnering with families enables person-centred care)	*“They’re the ones that [Family of PLWD] spend the majority of time with that person [PLWD]. And you can get a lot of information from them around what is normal*,* what is not normal*,* and strategies as well. Sometimes they’ll sort of say*,* ‘This is the particular routine they follow and this doesn’t happen and this happens’. So*,* identifying routines is a really good strategy and I often sort of put that into the behaviour care plans or into my notes quite explicitly”.* (Nursing_8 months in current role_#10) *“The thing that comes to mind the most prominently is probably utilising family members*,* even though it’s probably the most cliché thing. I don’t know*,* for me*,* that just resonates the most. It seems to help heaps with a face that can be recognised if they’re present*,* or if there’s a phone call or something”*. (Nursing_1 month in current role_#5) *“I’ve showered him twice but I’ll do it again today. I don’t think he’s had one since Tuesday… I did say to the nurse on charge that night that he’s not incontinent. He doesn’t really need pull ups. I know sometimes he has a problem remembering how to urinate*,* so maybe they just thought it was easier to put pull ups on him. I’m not really sure. But they haven’t after that”.* (Wife of PLWD_#4)
(Multidisciplinary teamwork and communication between staff facilitate holistic care)	*“The behaviour huddles we have found are amazing because we can see different lenses for people. So*,* for myself*,* I come from a nursing background*,* whereas mental health*,* the liaison there is very good at identifying*,* ‘Well*,* they’ve started this new medication*,* these are the side effects’. And then we have our neuropsych[ology] as well*,* so they’re seeing it more from a psychological perspective. So those huddles… Anyone you speak to*,* they are a really*,* really effective way of understanding. I think getting that understanding of what’s going on*,* rather than just responding to what’s happening to that person”*. (Nursing_8 months in current role_#10) *“I think it’s just the handover is the priority number one. It has to be done correctly. You can prevent all the injuries*,* all the code greys [emergency responses to aggressive behaviour] and all of that. That’s why a lot of people say our ward does do it well. I think because we sort of wait. We all take that time to talk to the people who are coming to do the extra care*,* to support us. And also*,* we want to support them… That takes a few minutes but it saves you trouble for the whole shift”.* (Nursing_10 years in current role_#3)
(Learning to care for PLWD comes with experience)	*“There is pretty much no teaching in undergraduate*,* postgraduate training around how to interact [with] or support a person with dementia. We might learn about what dementia is*,* but not actually how to care for the person with dementia. So*,* not in formal training in either of those disciplines*,* I can say confidently. And then*,* once you start working*,* it’s just real time experience*,* there’s no specific teaching”.* (Medical_6 months in current role_#8) *“Through Dementia Australia and the University of Tasmania*,* there’s lots of online training courses now*,* which keep you up-to-date with what the latest evidence shows for these patients. A lot of them are very community-based for obvious reasons”*. (Occupational therapy_5 years in current role_#11) *“It was empowering the junior staff*,* and I think that’s important. I think there’s a lot missing from their education in that actual side of things*,* I think they know clinical stuff really well*,* but it’s actually the behaviours they really struggle with”.* (Nursing_7 months in current role_#2) *“It’s giving the staff some confidence in using those strategies and tactics… and being ok about ‘Yes*,* he’s unsteady*,* but let’s not stand directly beside him because that clearly upsets him and increases his falls risk rather than decreases [it]. And let’s just give him his own personal space’. So*,* it’s about giving them the confidence to implement some of the strategies we suggest and also being open to role modelling those as well”.* (Nursing_8 months in current role_#10) *“I think the training needs to focus on understanding that person*,* understanding the disease*,* understanding that it’s a progressive disease*,* not a treatable one and focusing on what we can do*,* not to change behaviours*,* but to manage them within the setting. So*,* it’s not about trying to stop things happening*,* it’s about how can we respond and react in a way that minimises harm for both the patient and the staff… I know education isn’t the solution to everything*,* but I think in this case*,* until people really understand dementia and what it does to people*,* it’s hard to have that empathy and that patience with someone who is presenting with those challenging behaviours”*. (Nursing_8 months in current role_#10)
**Factors inhibiting quality care provision for PLWD**	
(Responsibility for understanding the needs and preferences of PLWD is unclear)	*“Also*,* whose responsibility is it because a lot of the time*,* the expectation is on nursing staff and they are just so busy…. I don’t think that’s entirely clear whose responsibility it is… This is where the difficulty lies*,* as if it’s the responsibility of one person and that person’s not available*,* the system falls apart. If responsibility is on everyone*,* then it gets murky as to who’s going to do what… I think we should all be doing it*,* whoever’s in touch with the family*,* if those needs and preferences haven’t been sorted and someone’s going to be in touch with the family*,* well then that’s part of the conversation you have with family”.* (Nursing_1 year in current role_#9)
(Current tools to understand and integrate the needs of PLWD are surface-level or reactive)	*“It might be that I’m going to see a patient that has made a change in escalation and behaviour and I’ve got just some prompts right up on the bedside*,* which can actually really help me in that de-escalation situation. It’s not something on the computer that I’m trying to find. It’s right there in front of you… It gives you just some cues to just kickstart some conversation… If I was to do an audit*,* it wouldn’t be done as much as I would prefer”.* (Nursing_1 year in current role_#9) *“The Sunflower tool is just a really quick snapshot*,* whereas the Behaviour Care Plan is*,* I guess it’s a more comprehensive plan that’s created actually through a behaviour meeting”.* (Nursing_1 year in current role_#9) *“They’re very time consuming and they take a lot of resources. So*,* when we do these behaviour care plans properly*,* it involves either a geriatrician or a senior trainee*,* a senior nursing member from that ward*,* risk staff*,* a neuropsychologist*,* often a member from the consultant liaison psychiatry team and a cognition team. So*,* you end up having a huge number of resources put into that document*,* which is why they are so amazing*,* but it’s just not practical to do that for every person*,* which is why we pick off the most challenging patients to do that. But by far they are the most helpful documents going around for dementia care*,* if they get read and applied properly. But often they’re in the notes and they’re embedded deep and people don’t read them*,* they don’t know they’re there… But patients who don’t have behaviour care plans*,* I’m playing catch up*,* everything’s reactive”.* (Medical_6 months in current role_#8) *“I think they ask*,* ‘Do you have dementia?’ or something. I can’t remember exactly what they ask and I can say*,* ‘Yes she does’*,* but there’s no further questioning about what are her needs*,* what type of dementia does she have*,* what do we need to know*,* that sort of thing. There’s no way to record any of that information”.* (Daughter of PLWD_#2)
(Formal communication channels are lacking between hospital staff and support people)	*“I think we have to work at different ways of communicating because there are carers involved and we can’t always be there”.* (Son of PLWD_#6) *“It was just hard the other night when the nurse rang me about his medication and then [husband’s name] hung up and because it’s an unknown number I couldn’t call back… There’s no number to ring. I can’t just ring and get through to the nurses”*. (Wife of PLWD_#4) *“Some formal way of communicating… I’d feel better with written because if I talk to somebody over the phone*,* sometimes people are distracted or they don’t hear or they don’t take it in*,* but if there was a chain of communication back and forth where you could raise a concern and have an answer back*,* that would be helpful… Ideally you’d want something every day because presumably they go in an see the patients every day*,* but if that’s not possible*,* is it possible to email something? Even if there was a book or something that you could write in*,* something that stays at her bed or something where you might be able to say*,* ‘Hey*,* can you keep an eye on her food intake today?’”* (Daughter of PLWD_#2) *“I didn’t even know he was being brought to the ward the next day… That was the only bit of lack of communication that I’ve had*,* that I would’ve liked to have known where he is and what’s being done with him”.* (Daughter of PLWD_#5)
(Lack of staff and time impact the quality of care received by PLWD)	*“Patients with dementia need time. They need someone to listen*,* and if you keep fobbing them off*,* especially with the patients with BPSD [behavioural and psychological symptoms of dementia]… it starts to escalate. I can literally see it”.* (Nursing_10 years in current role_#3) *“I talk to the patients themselves*,* and I think just trying to can be difficult some days*,* but try to find that time to talk to people. We rotate quite regularly and I find at night I like to take however long it takes to settle all my patients for the night. I’ll spend as much time as they need and work my way around*,* so I’m often still settling them quite late*,* but it gives me the opportunities to talk to them*,* ask questions*,* and I find that they settle and sleep better because they’ve had that one-on-one time”.* (Nursing_7 months in current role_#2) *“The extra resources like RUSONs [Registered Undergraduate Student of Nursing] or health assistants or PCAs [Personal Care Assistants] and those type of things definitely allow those patients to have the one-to-one time which could help with the filling out of paperwork or talking about*,* doing those sunflower things… they’re the people that can actually help with that. It’s awful and you wish you had time to do it*,* but we just don’t have that time”*. (Nursing_4.5 years in current role_#4) *“They’d assigned somebody to her so that when I wasn’t there*,* every time she went to get up or something*,* there was somebody there to say*,* ‘Hey*,* it’s okay*,* you’re in emergency [department]’… Which was really good because that’s what I was worried about… It was comforting for me to see in the morning when I got there that somebody had been keeping an eye on her”.* (Daughter of PLWD_#2)
**Considerations for future improvements to understanding and integrating the needs and preferences of PLWD**	
(Importance of a pre-emptive, rather than reactive solution)	*“Just having something to refer to with these needs and preferences*,* because we all need that information in different ways and it would be amazing to have access to that”.* (Occupational therapy_5 years in current role_#11) *“It’s actually being prepared when they walk in the front door*,* knowing what those preferences are”.* (Nursing_1 year in current role_#9) *“If that something could be accompanied*,* so you could read some background about him. Because you’re not going to get information from him. He can’t remember… To have a copy of this*,* if you went off to hospital again*,* would give you an idea of the person you’re dealing with”.* (Wife of PLWD_#8)
(Hospital admission kits should contain both information about PLWD and their familiar items)	*“Well it definitely*,* definitely helped her. As soon as I took the photos out of the bag and put them up*,* she was there naming everybody in the photo. So that definitely orientated her. Yeah fairly quickly. And*,* as I said*,* it’s a point of conversation*,* it gives the staff something to converse about*,* if they can see a photo there”.* (Daughter of PLWD_#9)
(Information about PLWD should be readily accessible)	*“Whether we had a tool or something that everyone could use and could access*,* that everyone can see what’s already been established and what still needs to be done next”.* (Occupational therapy_5 years in current role_#11*)*
(Variation in views regarding when hospital admission kits should be created)	*“It really has got to start from the community and has to be pre-emptive rather than reactive. So*,* having something that family members*,* loved ones can put together in the community*,* when the person is relatively well and stable*,* that can then provide all of that key information*,* right from the first moment that person enters the healthcare system*,* with the ambulances and the emergency department. But there are obviously challenges with that*,* with older people and their ability to utilise technology*,* and to have time to do this*,* and having resources available to help support that to occur in the community in the first place”.* (Medical_6 months in current role_#8) *“I think admission into the ward would be a great opportunity to go through things with the family”.* (Daughter of PLWD_#2) *“I could have filled something in easily while I was waiting there… On reflection*,* I spent a lot of downtime there*,* reading emails and catching up on social media”.* (Son of PLWD_#6)
(The utility of a hospital admission kit is likely to be limited by the advancement of dementia)	*“The ones [information packs] that you get from the nursing home might have a bit of information about how they ambulate around the ward or maybe has some of the things that they like to do. But for the most part*,* the reason they’ve presented to hospital in these situations is either delirium*,* which is not their baseline*,* or something else that’s exacerbated their dementia and has resulted in a hospital admission. So*,* it’s a bit hard… the information that is in those packs isn’t always how they’re presenting to us”*. (Nursing_4.5 years in current role_#4) *“He was a great golfer and loved his golf. Very proud of his hole-in-one. Shows everybody his plaque. In the last couple of months*,* couldn’t care less about that either. If I took his trophy in or anything*,* it wouldn’t mean anything to him. Where once upon a time he was very proud of it”*. (Wife of PLWD_#8)

Key: PLWD– People living with dementia

### Key principles of quality care for PLWD in hospital

#### Understanding and integrating needs improves patient care

All staff acknowledged the importance of understanding and integrating the needs of PLWD, highlighting that it helps to humanise patients, bring a sense of routine and familiarity to the hospital environment, engage them in their care, facilitate discharge planning, provide appropriate reassurance, de-escalate situations and be more proactive in terms of avoiding escalation of behaviours associated with dementia, thereby reducing the need for medical intervention and preventing or reducing patient harm.

Both staff and support people recounted several examples in which understanding and integrating patients’ needs worked well. These generally involved accommodating patients’ usual routines, encouraging independence, providing reassurance and allowing them to engage in activities of interest to them or tasks associated with their previous occupations, often utilising resources available on the ward.

Both staff and support people highlighted that there is no ‘one size fits all’ approach to caring for PLWD and that care needs to be individualised in order to respond to their behaviour as effectively as possible. However, it was also acknowledged that there were occasions where certain behaviours could not be prevented.

#### Partnering with families enables person-centred care

Staff members reported that the most efficient way to get to know patients and understand how to care for them in a resource-limited environment is often by engaging with family members rather than only talking to PLWD themselves. This is particularly the case if the cognitive presentation is different from their baseline (e.g., delirium) or in the setting of advanced dementia. Staff also acknowledged that while families generally know PLWD best, nursing home staff and primary care physicians could also provide useful background information, particularly if families were not involved in caring for PLWD at home. Staff recalled receiving very comprehensive information from nursing homes, particularly if the home had developed a behaviour care plan for the PLWD.

Staff emphasised the importance of speaking with families (or obtaining information from other sources) as soon as possible in order to incorporate strategies identified through past experience into current care plans, as well as to assist with forward planning / discharge planning. Limited availability of family members and the PLWD not speaking English made it difficult to obtain information, but one staff member reported that the family of PLWD usually made a significant effort to be available. Staff noted that COVID-19 acted as a barrier to obtaining information from families. Prior to COVID-19, staff could easily ask family members questions while they were visiting, whereas it often required multiple phone calls in the presence of visitation restrictions.

Directly involving family members in the care of PLWD was also seen as important as a de-escalation strategy, with staff acknowledging that family members can provide a sense of familiarity and often know how best to reassure them. Staff were also grateful for the extra physical assistance (e.g., repositioning in bed).

Support people also emphasised the importance of their physical presence in hospital, describing helping their family members with showering, feeding and dressing, as well as providing reassurance, noting that it is often difficult for PLWD to be left alone. Being physically present also enabled them to provide real-time information to staff. Support people recounted a number of situations in which their presence helped to ensure that appropriate care was provided.

#### Multidisciplinary teamwork and communication between staff facilitate holistic care

Staff emphasised that multidisciplinary teamwork and good communication was key to ensuring that PLWD receive the best care possible. They noted the holistic approach and multidisciplinary nature of good care plan development. Staff also acknowledged that COVID-19 further enabled multidisciplinary teamwork as meetings took place online and were easier for people to attend.

While many staff members favoured verbal communication, the importance of written communication (e.g., in the medical record or shift notes) was also highlighted to reduce duplication of effort. However, staff noted that, given the time taken to read all potentially relevant information as well as the time required to locate it, verbal communication can often be the most efficient as it enables staff to prioritise the most important information. Staff considered handover to be particularly important, arming staff with up-to-date information and strategies prior to seeing PLWD and also acknowledged that the more people communicate, the more knowledge staff accumulate, thereby improving care over time.

#### Learning to care for PLWD comes with experience

Staff highlighted the lack of formal training they had undertaken around caring for PLWD, instead learning from others and through experience. While some staff members specifically mentioned dementia modules offered by Dementia Australia and the University of Tasmania, others were not aware of any formal training opportunities. Some staff described conducting their own self-directed learning based on areas of particular interest. 

Staff also reported attending workplace-based education and study days prior to COVID-19 and suggested that these types of educational activities were particularly useful for newer staff, but also served as refreshers for others.

Some staff described modelling specific strategies to other staff in order to build their confidence in caring for PLWD.

While most staff reported that they felt equipped with a toolkit of strategies to care for PLWD, some did not, suggesting the need for more education, particularly for new staff, whilst also acknowledging the value of refreshers for older staff. They suggested that education should focus on behaviour management, as well as building capacity for nursing staff to take on specialist roles.

### Factors inhibiting quality care provision for PLWD

#### Responsibility for Understanding the needs and preferences of PLWD is unclear

While the various disciplines and team members involved in developing behaviour care plans were clear, responsibility for gathering additional information from families was not. Support people reported that they did not know to whom they should communicate their knowledge of the PLWD they were supporting to ensure that information would be passed on and implemented. Some staff suggested that responsibility for understanding and integrating the needs of PLWD and completing relevant tools should be clear, but should not fall to one person or discipline. One staff member suggested that responsibility for gathering information should fall to whoever contacted the family first, whether that be medical, nursing or allied health staff.

#### Current tools to understand and integrate the needs of PLWD are surface-level or reactiv 

Staff described two main tools for understanding and integrating the needs of patients with dementia– namely, the Sunflower tool and Behaviour Care Plans. The Sunflower tool was developed by the Agency for Clinical Innovation (New South Wales) and visually displays a person’s preferred name and up to 9 other areas including important people, past occupation and hobbies [23]. Behaviour Care Plans are locally developed clinical documents that are completed for those identified as potentially benefitting from them that focusses on assessment regarding changed behaviours, potential behaviour triggers, and successful and unsuccessful strategies [24]. Staff had mixed opinions about the Sunflower Tool, but largely reported that it was only useful at a surface level. While the tool provided some conversation topics that could help to build rapport or potentially de-escalate a situation, staff felt that it did not contain meaningful strategies for supporting PLWD. Staff appreciated the prominence of the tool at the bedside, highlighting that while the information may or may not help to de-escalate a situation, it was easily visible at the point of care provision. Some staff acknowledged that the tool was often not completed or still displayed information about a previous patient and they were not stored for potential future admissions. They also reported that families often noticed the Sunflower tools and offered to help the nursing staff complete them but this was not possible during COVID-19.

Conversely, Behaviour Care Plans were seen as very useful due to their comprehensive nature and the practical strategies they contained. However, their completion was seen as very reactive. The significant time and resources required to develop them were also highlighted, with staff noting that this limited their use to only the most challenging patients. Staff reiterated the need for a tool that could be completed prior to hospital admission to ensure that all patients could benefit from tailored strategies that work to integrate their needs. Difficulties accessing the Behaviour Care Plans and poor awareness of their existence limited their use, which staff identified as a missed opportunity.

Neither Behaviour Care Plans nor the Sunflower tool was mentioned by any participating support people. Support people reported that the extent to which staff asked about the needs of PLWD varied. They reported providing information about their family members to hospital staff on an ad-hoc basis, either because a staff member asked about a particular need or support people noticed that a particular need was not being met.

#### Formal communication channels are lacking between hospital staff and support people

While support people did not report any difficulties voicing the needs of their family member once they had the opportunity to do so, opportunities to communicate with staff were often limited. Support people expressed a need for more formalised communication processes in order to stay informed about their family member’s care and assuage any uncertainty, particularly when they were unable to visit.

Support people emphasised the importance of closed-loop communication to ensure that requests have been actioned. Suggestions for improvement included scheduling regular updates (either via phone or email), having one point of contact (i.e. a patient liaison) with a direct number to call and implementing communication books.

#### Lack of staff and time impact the quality of care received by PLWD

Staff described how competing demands within the hospital environment limited the time they had to understand and integrate the needs of patients with dementia, including spending adequate time with patients and completing and updating available tools (e.g. Sunflower tool and behaviour care plan). It was noted that the responsibility for completion of the Sunflower tool frequently fell to nursing staff. Staff also mentioned that if the behaviour care plan had been completed, they often lacked time to read the information that had been documented and subsequently incorporate any strategies into care.

However, staff acknowledged the importance of spending time with patients and the benefits when they were able to take the time to do so.

While staff and support people reported the benefits of having additional staff available, including allowing one-on-one time with patients, completion of tools and identification of patients who may require specialised input, staff highlighted that they do still require adequate knowledge around working with patients with dementia in order to add value to the team.

### Considerations for future improvements to understanding and integrating the needs and preferences of PLWD

#### Importance of a pre-emptive, rather than reactive solution

Staff expressed a need for some kind of solution, like the proposed hospital admission kit, in order to make care less reactive, spend less time trying to source information from other staff and families and allow more time for patient care and streamline discharge planning.

While most support people had not previously considered the idea of a hospital admission kit, they could see the value of doing so, feeling it could assist staff with rapport building and facilitate the provision of patient-centred care.

#### Hospital admission kits should contain both information about PLWD and their familiar items

Staff suggested that a hospital admission kit could include information about a person living with dementia and familiar items and ideally have this ready in case of a hospital attendance. Staff suggested including information to assist in getting to know patients and build rapport, as well as how to help support them in hospital. Specific suggestions from both staff and support people included: routines (e.g. what a normal day looks like and how and when they take their medication); religion; languages spoken; usual roles within the home; personality traits; stage of dementia; common behaviours (e.g. wandering); concerning behaviours (e.g. violent or aggressive tendencies); triggers for escalation; level of assistance required for various activities (e.g. dressing); level of continence; meaningful strategies to support them that have been effective in the past (as well as those strategies that were not effective); preferences for their general environment; main contacts and carers (including names of surrogate decision makers); important people in their life; likes and dislikes (e.g. food); hobbies / interests; pets; and previous occupations.

Staff also mentioned a number of physical items that could be useful to include, highlighting that as long as items are safe for the patient and not too loud, they should bring as much of the home environment into hospital as possible to foster a sense of familiarity. Suggestions included: photographs (e.g. of travels or family); items that represent their passions (e.g. football scarf or trophies); things that they use on a daily basis (e.g. electric shavers, hearing aids, glasses and iPads or other electronic devices); things to keep them busy (e.g. notebook and pen, magazines, newspapers, playing cards, fidget toys, puzzles, music and colouring books); favourite foods and drinks; religious items of significance (e.g. rosary beads); orientation-related items (e.g. clocks or calendars); and things to help them feel calmer and more comfortable (e.g. their own clothes, pillows, blankets and weighted toys). Two staff members expressed concern that patient items could be lost or damaged during their stay and some support people who had previously brought in personal items had also taken this into consideration when choosing what to bring. One support person was also worried about making the environment look too permanent, which influenced the items they chose to bring for their family member.

Although they had not put a ‘kit’ together prior to admission, some support people had brought in personal items for their family members and seen an immediate benefit. However, the integration of these items was largely by support people rather than staff (i.e. family members looking through photos with PLWD).

#### Information about PLWD should be readily accessible

Staff emphasised the need for the informational component of the hospital admission kit to be easily accessible in multiple settings (i.e. in the community setting for completion and in the hospital setting for utilisation and updating), but were unsure about how this could be coordinated. Support people suggested developing a form that was easily accessible online and via hard-copy to suit individual preferences. One participant also suggested that it could be completed via an app. They suggested that the form should include tick-boxes, space for photos and should be customisable (i.e., only sections relevant to the PLWD need be completed). While some staff stated a preference for a hard copy document, they acknowledged that it could easily get lost amongst other paperwork. Other staff suggested that it would need to be electronic so that it could be easily updated and travel with the patient across settings. However, they recognised the complexity of this format as not all staff can access the electronic medical record (EMR) and health services often use different systems, thereby limiting transferability. They also mentioned that if the hospital admission kit was to form part of the EMR, an alert would need to appear so that staff are aware that this additional information is available. Other suggestions included using the Australian government-developed, My Health Record, to enable access across settings, with a notification of this being visible within the local EMR [25]; use of a medical alert bracelet; or personal card using QR codes.

#### Variation in views regarding when hospital admission kits should be created

Some staff members suggested that the creation of a hospital admission kit should commence as early as possible (i.e. when someone is first diagnosed with dementia) as they are more likely to be relatively well and stable and can contribute to personalising the information included. Staff suggested that this process could be supported by a primary care physician, staff members working in an aged care facility or another support person who is familiar with dementia (either through personal or professional experience e.g. peers via Dementia Australia). They also noted that this information could then be updated as things change over time. Alternatively, it could be conducted at key moments of change (e.g. when someone is undergoing an assessment for formal social supports).

Conversely, some support people suggested that it would be good to have a conversation with a staff member and complete the informational component of a hospital admission kit when a PLWD is being admitted to the ward. They also noted the potential time available to complete this task when waiting to be seen in the Emergency Department.

#### The utility of a hospital admission kit is likely to be limited by the advancement of dementia

Both staff and support people acknowledged that the informational component of a hospital admission kit may not be representative of how a PLWD presents in hospital if they have deteriorated either prior to or following admission, such that family members may not know how to support them anymore.

While support people could easily think of topics to include in the informational component of a hospital admission kit, some struggled to think of physical items that would be useful (beyond photos) because their family members had either lost interest in the activities they had previously enjoyed or lacked the attention span to engage with them. They noted that this could make it difficult to put together the physical aspect of a hospital admission kit as the benefits of certain physical items may differ as dementia progresses. However, they could specify items that would have been useful to include prior to the advancement of dementia or onset of other physical limitations (e.g. deterioration in vision or hearing alongside dementia).

## Discussion

We found that participants were consistent in highlighting the importance of understanding and integrating the needs of PLWD into clinical care provision and that communication helped people supporting a PLWD to partner with the multidisciplinary healthcare team to provide high quality care. Participants reported that a lack of time, staff and training, environmental constraints and a lack of opportunities for families to communicate with staff were barriers to providing quality care. Participants felt that currently used tools were not fit for purpose, but were supportive of the concept of having materials pre-prepared in case of the need of hospital attendance. Hospital staff and people supporting PLWD had a number of different suggestions for what such a ‘toolkit’ might entail. However, there were substantial variations in the nature of these suggestions, supporting our planned next steps to enter into a formal co-design process.

The high priority that participants placed upon incorporating the needs and preferences of PLWD into hospital care provision is supported by the results of previous studies [4, 6, 7, 13–15, 17, 26, 27]. Previous work has also highlighted the absence of effective interventions to facilitate this integration [6, 13, 15]. Participants in our study reported that they felt locally-used tools were not fit for purpose. This is consistent with existing literature examining a broader scope of widely available tools, (e.g. healthcare passports) further highlighting the need to consider both the design and implementation aspects of potential solutions [18, 19].

Conducting this study during the COVID-19 pandemic highlighted that processes involving face-to-face communication with family members to understand needs and preferences, are weak and easily disrupted. As such, ideal solutions will need to be functional in the absence of family members being physically present. As highlighted by participants, the designed solution will likely need to pre-empt rather than react to an acute deterioration necessitating hospital attendance. An important part of the design process will involve ensuring the responsibility to create, update and link this to healthcare workers is clearly assigned. PLWD, those supporting them, primary care providers, residential care homes, advocacy groups (e.g. Dementia Australia) and hospital staff were all identified as potential candidates. The optimal time to complete and update this information also requires further input. Suggestions included at the time of dementia diagnosis, at primary care visits, supported by family at home or on hospital admission/discharge. All of these epochs have specific advantages and disadvantages but do not necessarily need to be mutually exclusive. Similarly, the actions performed by staff to meet the hospital care needs of PLWD once they use the toolkit needs to be considered. Healthcare provider workflows will need to be designed in such a way that material within the toolkit will be sought and consistently incorporated into clinical care with clear understanding of roles and accountability.

Additional design considerations include the need for the hospital admission kit to be somewhat fluid or ‘living’ to accommodate evolving conditions, behaviours, needs and preferences, which presents a different design challenge, than with a ‘set and forget’ tool. Furthermore, staff highlighted that a significant amount of information would be useful to know, whilst also repeatedly reporting that they were time poor. Consequently, the quick and timely translation of key and/or filtered information will need to be considered in the design.

This study has a number of strengths and limitations. We were able to obtain the perspectives of a range of people supporting PLWD, including spouses and children. We also obtained perspectives from hospital staff over a range of clinical disciplines and experience. However, an important limitation was our inability to include PLWD. Although including this important perspective was an aim of this study, those PLWD who were potentially eligible did not remain medically stable enough post hospital discharge to be able to participate. We chose to recruit from the acute hospital setting as we wanted to optimise the ability of a PLWD being able to remember their recent hospital experience. Future work should consider recruiting people in the community setting to examine the feasibility of this approach. However, our experience highlights the clinical need and a range of design challenges associated with developing a toolkit for such a vulnerable and clinically unstable group. Reassuringly, many of the themes we identified in our study were consistent with those reported by a previous review of seven qualitative studies that did include PLWD [14]. A further limitation of our study was our focus on participants’ experience at a single clinical site and in those who speak English. Therefore, the generalisability of our results is unclear.

## Conclusions

We found that hospital staff and those supporting PLWD felt that integrating the needs and preferences of PLWD into hospital care was important. The concept of a pre-prepared ‘toolkit’ that was ready in the case of a need to attend hospital was felt to be valid and potentially helpful. More work is required to design aspects such as format, content and the workflows needed to generate accountability and reliability in creating, updating and incorporating it into hospital care.

## Electronic supplementary material

Below is the link to the electronic supplementary material.

Supplementary Material 1

Supplementary Material 2

## Data Availability

The transcripts from this study include potentially identifiable / confidential participant information and are not publicly available. Further information will be available from the corresponding author on reasonable request.

## Abbreviations

PLWD: People living with dementia
EMR: Electronic medical record
ACE: Acute Care of the Elderly

## References

[1] Australian Commission on Safety and Quality in Health Care. Evidence for the safety and quality issues associated with the care of patients with cognitive impairment in acute care settings: a rapid review. 2013.

[2] Fogg C, Griffiths P, Meredith P, Bridges J. Hospital outcomes of older people with cognitive impairment: an integrative review. Int J Geriatr Psychiatry. 2018. 10.1002/gps.4919 PMC6099229 29947150

[3] Greenfield S, Kaplan S, Ware JE. Jr. Expanding patient involvement in care. Effects on patient outcomes. Ann Intern Med. 1985;102(4):520–8. 3977198 10.7326/0003-4819-102-4-520

[4] Turner A, Eccles FJ, Elvish R, Simpson J, Keady J. The experience of caring for patients with dementia within a general hospital setting: a meta-synthesis of the qualitative literature. Aging Ment Health. 2017;21(1):66–76. 26553275 10.1080/13607863.2015.1109057

[5] National Institutes on Aging. Going to the Hospital: Tips for Dementia Caregivers 2020 [Available from: https://www.nia.nih.gov/health/going-hospital-tips-dementia-caregivers

[6] Houghton C, Murphy K, Brooker D, Casey D. Healthcare staffs’ experiences and perceptions of caring for people with dementia in the acute setting: qualitative evidence synthesis. Int J Nurs Stud. 2016;61:104–16. 27343469 10.1016/j.ijnurstu.2016.06.001

[7] Gwernan-Jones R, Abbott R, Lourida I, Rogers M, Green C, Ball S, et al. The experiences of hospital staff who provide care for people living with dementia: A systematic review and synthesis of qualitative studies. Int J Older People Nurs. 2020;15(4):e12325. 32412167 10.1111/opn.12325

[8] National Institute for Health and Care Excellence. Transition between inpatient hospital settings and community or care home settings for adults with social care needs. 2015.

[9] Alzheimer’s Society. Going into hospital 2020 [Available from: https://www.alzheimers.org.uk/get-support/help-dementia-care/going-into-hospital

[10] Dementia Australia. Hospital Care for People Living with Dementia. 2019.

[11] Australian Commission on Safety and Quality in Health Care. National Safety and Quality Health Service Standards. 2020.

[12] Burgstaller M, Mayer H, Schiess C, Saxer S. Experiences and needs of relatives of people with dementia in acute hospitals—A meta-synthesis of qualitative studies. J Clin Nurs. 2018;27(3–4):502–15. 28639361 10.1111/jocn.13934

[13] Digby R, Lee S, Williams A. The experience of people with dementia and nurses in hospital: an integrative review. J Clin Nurs. 2017;26(9–10):1152–71. 27322590 10.1111/jocn.13429

[14] Reilly JC, Houghton C. The experiences and perceptions of care in acute settings for patients living with dementia: a qualitative evidence synthesis. Int J Nurs Stud. 2019;96:82–90. 31345443 10.1016/j.ijnurstu.2019.04.018

[15] Røsvik J, Rokstad AMM. What are the needs of people with dementia in acute hospital settings, and what interventions are made to Meet these needs? A systematic integrative review of the literature. BMC Health Serv Res. 2020;20(1):1–20. 10.1186/s12913-020-05618-3 PMC7412803 32767987

[16] Siostrom K, Snowdon D, Sriamareswaran RK, Law YM, Jolliffe L, Moran C. Experiences of healthcare staff caring for hospitalised people with delirium: a qualitative systematic review. Age Ageing. 2024;53(7). 10.1093/ageing/afae159 39078153

[17] Anantapong K, Davies N, Sampson EL. Communication between the multidisciplinary team and families regarding nutrition and hydration for people with severe dementia in acute hospitals: a qualitative study. Age Ageing. 2022;51(11). 10.1093/ageing/afac230 PMC9701106 36434801

[18] Beattie F, Kerr L, Larkin J, Cawley D. The components of personal passports for people living with dementia in an acute healthcare setting: an integrative review. J Clin Nurs. 2022;31(13–14):1907–20. 33555640 10.1111/jocn.15702

[19] Clark E, Wood F, Wood S. Barriers and facilitators to the use of personal information documents in health and social care settings for people living with dementia: A thematic synthesis and mapping to the COM-B framework. Health Expect. 2022;25(4):1215–31. 35415955 10.1111/hex.13497 PMC9327869

[20] Van Manen M. Researching lived experience: human science for an action sensitive pedagogy. Routledge; 2016.

[21] Malterud K, Siersma VD, Guassora AD. Sample size in qualitative interview studies: guided by information power. Qual Health Res. 2016;26(13):1753–60. 26613970 10.1177/1049732315617444

[22] Ritchie J. Qualitative data analysis for applied policy research. In: Bryman A, R B, editors. Analyzing qualitative data. London: Routledge; 1994. pp. 173–94.

[23] State of NSW (Agency for Clinical Innovation). Sunflower. Sydney: Agency for Clinical Innovation. 2022 [Available from: https://aci.health.nsw.gov.au/__data/assets/pdf_file/0008/285380/ACI-Agedcare-CHOPs-Sunflower.pdf

[24] Aged Care Quality and Safety Commission. Behaviour support plans: A fact sheet for residential aged care providers.: Australian Government. 2021 [Available from: https://www.agedcarequality.gov.au/sites/default/files/media/fact-sheet-behaviour-support-plans.pdf

[25] Australian Digital Health Agency. My Health Record: Australian Government. 2024 [Available from: https://www.digitalhealth.gov.au/initiatives-and-programs/my-health-record

[26] Manietta C, Purwins D, Reinhard A, Knecht C, Roes M. Characteristics of dementia-friendly hospitals: an integrative review. BMC Geriatr. 2022;22(1):1–16. 35641899 10.1186/s12877-022-03103-6 PMC9158310

[27] Abbott RA, Rogers M, Lourida I, Green C, Ball S, Hemsley A, et al. New horizons for caring for people with dementia in hospital: the DEMENTIA CARE pointers for service change. Age Ageing. 2022;51(9):afac190. 36057987 10.1093/ageing/afac190 PMC9441201

