# Supplementary material for: Understanding and integrating the needs and preferences of people living with dementia in the inpatient setting: a qualitative study
Source: BMC Geriatr. 2025 May 15;25:342. doi: 10.1186/s12877-025-05932-7 (PMC12080157; doi:10.1186/s12877-025-05932-7)
Supplement: Supplementary file 1 — Supplementary Material 1 [file 12877_2025_5932_MOESM1_ESM.docx]

**Appendix 1 – Interview frameworks**

The interviews were semi-structured, allowing the interviewer to explore emerging themes and salient issues. The interview frameworks were as follows:

#### Staff

Introductory statement: People living with dementia often receive suboptimal care because of difficulties in communicating their needs and preferences. The aim of this project is to co-design a hospital admission kit that can be embedded into a revised Model of Care that helps clinicians to meet the needs and preferences of people with dementia who are admitted to hospital. Given that nursing staff / allied health staff play an integral role in the care of people with dementia in hospitals, we’re interested in exploring your thoughts about current practices, including what is done well and what could be improved, and the potential to implement a hospital admission kit.

Do you have any questions before we start?

1. Can you provide a brief introduction and outline your role in caring for people living with dementia, including how long you have been in this role?
2. What tools currently used within the organisation do you utilise to find out about a person living with dementia’s needs and preferences?
   1. *Are these valuable?*
3. Is there anything you have introduced into your own practice to help you identify these needs and preferences that you have found to be beneficial?
4. How do you integrate these needs and preferences into hospital care?
   1. How important do you think it is to integrate these needs and preferences?
5. What level of training and support have you received / do you receive on an ongoing basis to adequately care for people living with dementia?
   1. *Do you feel equipped (i.e. with a toolkit of strategies to care for someone with cognitive impairment)?*
   2. *Do you feel that you can readily identify someone who may have cognitive impairment or dementia if this information has not been made explicit during admission?*
6. From your perspective, what are the key issues that need to be addressed to ensure that the needs and preferences of people living with dementia are integrated into hospital care?
   1. *For patients?*
   2. *For support people?*
   3. *For hospital staff?*
7. Can you recall particular instances where understanding and/or integrating the needs and preferences of people living with dementia into hospital care worked well?
   1. *If so, what facilitated this process?*
   2. *Do you think it improved the patient experience? And if so, how?*
8. What kind of support would you need to successfully implement a hospital admission kit?

*Within the current system:*

- 1. *What are the barriers to achieving this?*
  2. *What are potential enablers?*

1. In an ideal world, how you would like to find out about a person living with dementia’s needs and preferences?
2. Are you interested in participating in a workshop to contribute to the design of a hospital admission toolkit for people living with dementia?

#### Support people

Introductory statement: We are researching the hospital care experience of people living with dementia. We would like to ask you some questions about your experience supporting a person with dementia. This will help us to understand how we might develop a toolkit to support people with dementia when they go to hospital and provide an improved care experience.

1. Can you tell me about the last time [they] were in hospital?
   1. *What went well?*
   2. *What didn’t go well / what was missing? What could have been improved?*
2. What parts of their existing home routine do you think would help them feel more supported when in hospital?
   1. *When they go to hospital, what do you think is different or missing compared to that routine?*
   2. *What is it about being in hospital that is challenging or doesn’t make them feel safe and supported?*
3. What have you noticed hospital staff do to support them?
   1. *What has been done well?*
   2. *What hasn’t gone so well / what has been overlooked?*
   3. *What are the opportunities for improvement?*
4. How did you prepare the person you support for this admission?
   1. *What works well?*
   2. *What would you do differently?*
5. How do you currently communicate the needs and preferences of the person you support to hospital staff?
   1. *What works well?*
   2. *What do you find challenging?*
   3. *What would make it easier to communicate their needs?*
6. How did the hospital help to prepare the person you support for this admission?
   1. *Did the hospital ask about their communication and support needs?*
      1. *If yes, what did they ask? How could this be improved?*
      2. *If no:*
         1. *What would you have liked to have been able to communicate about their needs?*
         2. *How would you have wanted the hospital to assist in preparing the person you support for this admission?*
7. If you had a magic wand, what would you change to make it easier to communicate their needs and preferences / ensure that their needs are met? *Is there anything that you wish existed that doesn’t exist at the moment to help you communicate the needs and preferences of the person you support?*
8. One potential solution that we’re thinking of developing is a hospital admission kit to help make sure that the needs and preferences of people with dementia can be integrated into hospital care. That kit could include a document where you can detail some information about likes and dislikes and routines / things that help keep people with dementia comfortable and supported and potentially some familiar objects ready to go in case they are admitted to hospital.

How do you think something like that would help you and the person you support? What do you think would be important to include? What might stop you from engaging with something like that? What would help you to engage with something like that?

1. Are you interested in participating in a workshop to contribute to the design of a hospital admission toolkit for people living with dementia?
